# Early stabilization of the uncemented Symax hip stem in a 2-year RSA study

**DOI:** 10.1080/17453674.2019.1709956

**Published:** 2020-01-13

**Authors:** Dennis S M G Kruijntjens, Lennard Koster, Bart L Kaptein, Liesbeth M C Jutten, Jacobus J Arts, René H M Ten Broeke

**Affiliations:** a Department of Orthopaedic Surgery, Research School Caphri, Maastricht University Medical Centre, Maastricht;; b Department of Orthopaedic Surgery, RSAcore, Leiden University Medical Centre, Leiden, the Netherlands

## Abstract

Background and purpose — The uncemented Symax hip stem has shown early proximal ingrowth as result of the BONIT-hydroxyapatite (HA) coating and the distal DOTIZE surface treatment. We evaluated 2-year postoperative radiostereometric analysis (RSA) migration of the Symax hip stem in THA patients. We also investigated the correlation between migration at 4 weeks and clinical outcomes after 2 years.

Patients and methods — Patients in a 2-year clinical follow-up single-centre RSA randomized controlled trial were randomized to 2 different cup designs. All 45 patients received a Symax hip stem. RSA migration patterns of the Symax hip stem is presented here as a single cohort. RSA examinations were performed postoperatively, but before weight-bearing, and subsequently after 1, 3, 6, 12, and 24 months. Clinical outcomes and radiographic evaluations were assessed 3, 6, 12, and 24 months postoperatively.

Results — During the first 4 weeks, the Symax hip stem subsided, rotated into retroversion, and translated posteriorly, after which the migration ceased and the prosthesis stabilized. All clinical outcomes improved from preoperatively to 2 years. There was no clinically or statistically significant correlation between subsidence and retroversion at 4 weeks and clinical outcomes after 2 years.

Interpretation — RSA evaluation of the uncemented Symax hip stem confirms that the design principles and coating properties lead to early stabilization of the stem, as early as 4 weeks postoperatively. There was no correlation between subsidence and retroversion at 4 weeks and clinical outcomes after 2 years. Based on the predictive potential of the RSA technique, we anticipate excellent long-term survival of this hip stem.

The uncemented Symax hip stem was developed as an optimization of the uncemented Omnifit hip stem (Stryker Orthopaedics, Kalamazoo, MI, USA) (Capello et al. 2009). The design considers the geometry of the stem, surface texture, and type and extent of the osseointegrative coating. Previous studies with histological and histomorphometric analyses on retrieved Symax hip stems have proven early bone ingrowth exclusively into the proximal part of the stem, as a result of the BONIT-hydroxyapatite (HA) coating (DOT GmbH, Rostock, Germany). A 2-year follow-up dual-energy X-ray absorptiometry (DEXA) study showed improved bone remodeling with the Symax hip stem compared with the Omnifit hip stem (ten Broeke et al. 2011, 2012, 2014). The current study is part of an RCT RSA study, assessing migration characteristics of 2 different uncemented cup designs (Trident HA and Trident Tritanium respectively; Stryker Orthopaedics, Kalamazoo, MI, USA) and the Symax hip stem with radiostereometric analysis (RSA). The focus of this study is on the migration pattern of the Symax stem in a single cohort, seen in the light of its specific optimized geometrical features, and in the light of the existing knowledge on mid-term outcome of this stem design. Several studies have shown that RSA can predict long-term prosthetic loosening based on 2-year postoperative follow-up data (Kärrholm et al. 1994, Valstar et al. 2005, Nieuwenhuijse et al. 2012).

We hypothesized that the design of the Symax hip stem leads to early stabilization, by 3 months postoperatively. The primary objective of this study was to evaluate early postoperative migration of the Symax hip stem with the use of RSA. The secondary objective was to investigate whether there is a correlation between migration of the stem at 4 weeks and clinical outcomes after 2 years.

## Patients and methods

This single-center RSA study was performed at the Maastricht University Medical Centre (MUMC), the Netherlands. It is a sub-study of an RCT RSA study comparing the uncemented Trident HA cup and the Trident Tritanium cup designs, presenting the migration results of the Symax hip stem, used in both groups, as a single cohort. The results of the acetabular cup migration will be reported in a separate manuscript. Enrolment took place between December 2011 and January 2015. Patients scheduled for uncemented primary total hip arthroplasty (THA), aged between 18 and 70 years, with BMI less or equal to 35 were eligible for this study. Exclusion criteria were bilateral hip complaints, impaired cognitive function, and use of medication or illness influencing bone metabolism (e.g., corticosteroids, bisphosphonates, osteoporosis, and metastasis).

### Surgical protocol

The posterolateral approach was used by 2 senior hip surgeons (RtB and JG), with transosseous reattachment of capsule and external rotators in all patients. Patients received 24-hour intravenous antibiotic prophylaxis (cefuroxime), deep venous thrombosis prophylaxis with low molecular weight heparins (nadroparin) for 6 weeks, and prevention of heterotopic bone formation with NSAIDs (indometacin) for 2 weeks. Full weight-bearing under the supervision of a physiotherapist was allowed from the first postoperative day, but only after the baseline RSA imaging was completed.

### Implant

**Figure 1.**
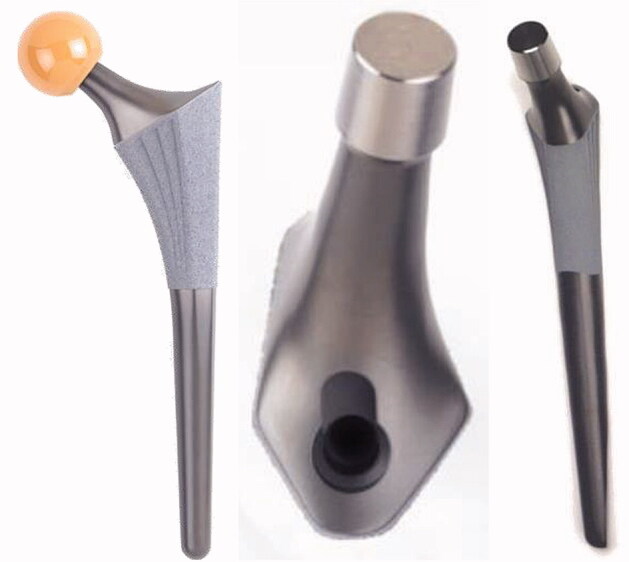
Design features of Symax hip stem, illustrating the anatomically anteverted proximal geometry, with the BONIT-HA coating; and the straight distal part with the DOTIZE surface treatment and a posterior chamfer.

The Symax hip stem is an uncemented design forged from Ti_6_Al_4_V alloy (CE 545074). The Symax design is an optimization of the uncemented Omnifit hip stem (Figure 1). For further details on geometry, surface treatment and coating characteristics, please refer to Szmukler-Moncler et al. (2001), Becker et al. (2004), ten Broeke et al. (2011).

Implanted cups were either the uncemented Trident HA (CE514410) or the uncemented Trident Tritanium (CE526088) acetabular cup, both in combination with an ultra-high molecular weight polyethylene X-3 insert (CE509982, Stryker Orthopaedics, Kalamazoo, MI, USA).

### RSA examination

**Figure 2.**
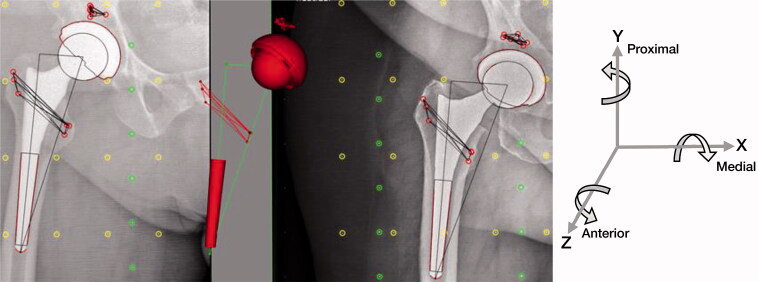
The Elementary Geometrical Shapes (EGS) model used to determine the migration of the Symax hip stem.

Baseline RSA examination was made 1 day after surgery, prior to loading of the operated hip. Follow-up RSA examination was made postoperatively at 1, 3, 6, 12, and 24 months. During surgery 6–8 tantalum markers (0.8 mm diameter) were inserted in the bone surrounding the hip stem. These markers form a rigid body that is the basis for RSA calculations. The rigid body consisted of at least 3 markers with a mean error (ME) of rigid body fitting below 0.35 mm and Condition Number (CN) below 120 (ISO 16087, 2013). All RSA examinations were acquired with the patient in a supine position over a uniplanar calibration box (Medis Carbon Box nr. 013, Medis Specials bv, the Netherlands). Migration of the Symax stems was calculated using the Elementary Geometrical Shapes (EGS) hip model from Model-based RSA software (Version 4.1; RSAcore, Department of Orthopaedic Surgery, LUMC, the Netherlands) (Figure 2) as described by Kaptein et al. (2006).

Translations and rotations were calculated using a coordinate system with its origin in the center of the 3D model in the baseline evaluation, and the X- and Y-axis parallel to the X- and Y-axis of the calibration box (Figure 2). Aligning the patient with the coordinate system of the calibration box allows for description of the migrations of prosthesis components using anatomical directions. Migration results from patients with a left-sided prosthesis were recalculated following the conventions as presented in the ISO standard guidelines (ISO 16087, 2013) in order to describe the migration in anatomical terms for a right-sided prosthesis. Migrations are reported as migration relative to baseline.

To determine the precision of the RSA set-up, a double set of RSA examinations during the same follow-up was acquired. Actual migration within the short time interval between the double examinations is expected to be 0, therefore calculated migration between these double examinations represents the measurement error. The mean error value represents the system bias, while the standard deviation (SD) is a measure for the precision of the measurements (Kaptein et al. 2006).

### Clinical evaluation

Clinical evaluations were performed preoperatively and postoperatively at 3, 6, 12, and 24 months. The evaluated clinical outcome parameters were the Harris Hip Score (HHS), the Oxford Hip Score (OHS), the Western Ontario and McMaster Universities Osteoarthritis Index (WOMAC), and the EuroQol-5D (EQ-5D). The EQ-5D index was calculated based on a Dutch value set, representative of the Dutch population with regard to age and sex. To facilitate further analysis the index was linearly rescaled on a scale of 0 to 100, with 0 the worst possible score and 100 the best possible score. Additionally, the EQ-5D VAS score was evaluated. Complications and adverse events were recorded during follow-up.

### Radiographic evaluation

Standing anteroposterior radiographs of the pelvis and axial radiographs of the operated hips were evaluated immediately postoperatively and at 3, 6, 12, and 24 months. Radiographs were evaluated as per “adapted Gruen zones” for signs of osseointegration, such as cancellous condensation (“spotweld formation”) and formation of distal reactive lines. In the “adapted Gruen zones”, zones 1 and 7 represent the coated areas, and zones 2–3 and 5–6 respectively the lateral and medial zones, equally divided around the non-coated part of the stem (ten Broeke et al. 2012).

### Statistics

Descriptive statistics of continuous variables were reported as means (SD) or (range) in case of skewness. Categorical variables are presented as frequencies. A paired samples t-test was used to compare migration between different follow-up moments. The Wilcoxon signed-ranks test was used as the non-parametric test to compare clinical outcome scores over time. Spearman’s rho correlation coefficients were determined to correlate Y-translation and Y-rotation at 4 weeks to clinical outcomes after 2 years. IBM SPSS for Windows 24.0.1 (IBM Corp, Armonk, NY, USA) was used for statistical data analysis, and p-values of < 0.05 were considered to indicate statistical significance. All results are reported with 95% confidence intervals (CI), if applicable, and clinical relevance will be discussed.

### Ethics, registration, funding, and potential conflicts of interests

Ethical approval was obtained from the local Institutional Review Board (MEC10-1-068 NL 33832.068.10). The study was registered in the ClinicalTrial.gov database (NCT01618084). Informed consent was obtained from each patient prior to surgery. The study was conducted according to the ethical standards of the Declaration of Helsinki of 1975, as revised in 2013 in Fortaleza (Brazil), and following Good Clinical Practice (GCP) and ISO 14155 guidelines. No conflict of interest was declared and no personal funding was received. Research grant was received from Stryker Orthopaedics (Kalamazoo, MI, USA). 

## Results

45 patients (22 female) were included, with a mean age of 59 years (30–70), and all patients were operated because of osteoarthritis, except 1 who was operated for avascular necrosis (Table 1).

**Table 1.** Baseline characteristics of included patients

	Female	Male	Total
Factor	(n = 22)	(n = 23)	(n = 45)
Mean age (range)	60 (44–70)	59 (30–70)	60 (30–70)
BMI (SD)	27 (4)	27 (3)	27 (3)
Osteoarthritis/avascular necrosis	22/0	22/1	44/1

### RSA evaluations

Due to not meeting the rigid body criteria, RSA results could not be calculated for 10 of the 45 patients. During follow-up several RSA acquisitions could not be used for RSA calculations, due to technical issues or over-projection of the bone markers by the hip stem. The number of the available migration calculations at each follow-up moment is denoted in Table 2.

**Table 2.** Number of patients per follow-up moment

Evaluation	Baseline	4 weeks	3 months	6 months	1 year	2 years
RSA analysis	35	33	32	32	31	31
Clinical	45	–	45	45	45	44
Radiographic	45	–	45	45	45	44

Mean migration of the 30 available RSA double examinations was –0.02 mm (CI –0.29 to 0.25) for X-translation, –0.01 mm (CI –0.25 to 0.23) for Y-translation, and –0.04 mm (CI –0.65 to 0.57) for Z-translation; –0.09˚ (CI –0.77 to 0.58) for X-rotation, 0.26˚ (CI –1.23 to 1.75) for Y-rotation, and –0.01˚ (CI –0.19 to 0.17) for Z-rotation. These results confirm that there is no bias in the RSA set-up.

At 4 weeks mean Y-translation was –1.0 mm (CI –3.4 to 1.4), mean Y-rotation (retroversion) was 2.4˚ (CI –2.2 to 7.0), and mean Z-translation (posterior translation) was –0.4 mm (CI –1.7 to 0.9). Migrations in the other directions were minor during follow-up (Figure 3). Subsidence was large (5.8 mm) initially in 1 patient, probably because of undersizing the stem; however, the position of this stem also stabilized after 4 weeks. This patient and the 13 patients who could not be evaluated with RSA after 2 years showed excellent clinical outcome scores at the time of their last RSA measurement as well as after 2 years.

**Figure 3a.**
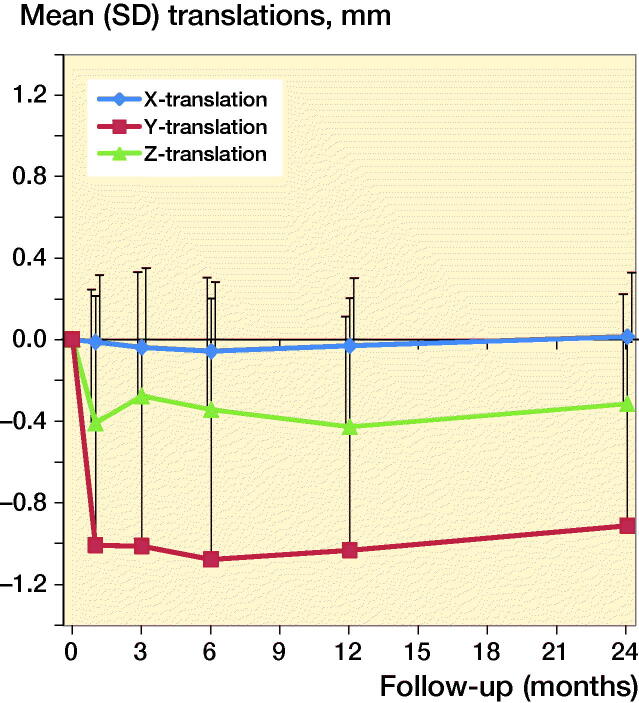
Mean translation results for the Symax hip stem.

**Figure 3b.**
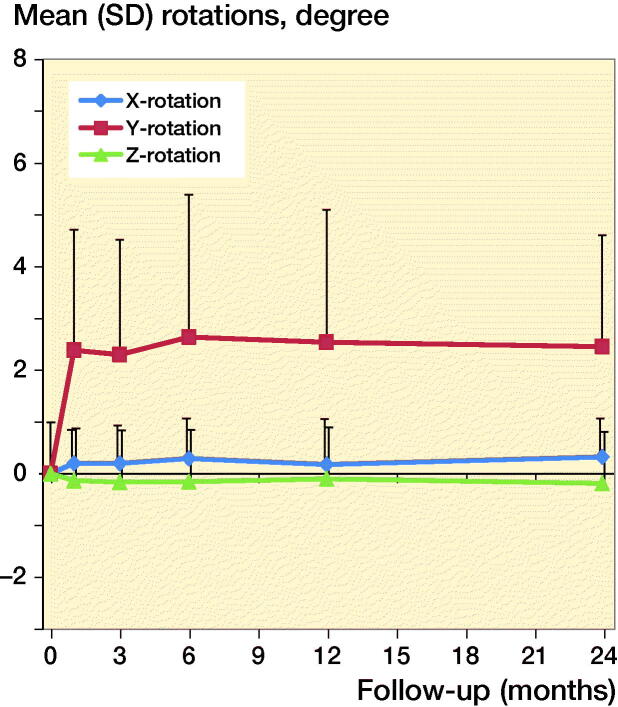
Mean rotation results for the Symax hip stem.

### Clinical results

All clinical outcomes improved clinically and statistically significantly from preoperative to 2 years (Table 3, see Supplementary data).

**Table 3.** Clinical outcomes as mean (CI) HHS, OHS, WOMAC, and EQ-5D over time

Score/subscore	Preoperatively	3 months	6 months	1 year	2 years
HHS	63 (33 to 93)	96 (82 to 110) ^a^	98 (91 to 105) ^a^	98 (90 to 107)	99 (92 to 105) ^a^
OHS	37 (22 to 53)	23 (7 to 39) ^a^	20 (6 to 34) ^a^	19 (5 to 33)	18 (5 to 31) ^a^
WOMAC					
Total	50 (13 to 86)	17 (–8 to 41) ^a^	13 (–13 to 39) ^a^	12 (–14 to 38)	10 (–14 to 34)
Pain	10 (2 to 18)	2 (–3 to 7) ^a^	2 (–4 to 7)	2 (–4 to 8)	1 (–4 to 7)
Stiffness	4 (0 to 8)	2 (–1 to 5) ^a^	2 (–1 to 5) ^a^	1 (–1 to 4)	1 (–1 to 4)
Physical functioning	36 (10 to 62)	12 (–6 to 30) ^a^	9 (–10 to 28) ^a^	9 (–10 to 27)	7 (–11 to 25)
EQ-5D					
Index	69 (42 to 96)	88 (68 to 107) ^a^	91 (72 to 110) ^a^	91 (72 to 110)	93 (75 to 111)
VAS	67 (26 to 108)	83 (56 to 109) ^a^	84 (58 to 110)	84 (55 to 113)	87 (58 to 116) ^a^

**^a^**Indicates Wilcoxon signed-rank test p-value < 0.05 compared with the preceding clinical outcome.

### Correlation RSA and clinical outcomes

There were no clinically or statistically significant correlations between Y-translation or Y-rotation at 4 weeks and any clinical outcomes after 2 years (Table 4, see Supplementary data).

**Table 4.** Spearman’s rho correlation coefficients (CC), with p-values, for correlation between Y-translation and Y-rotation at 4 weeks and clinical outcomes (HHS, OHS, WOMAC, and EQ-5D) after 2 years

	CC Y			CC Y
Score/subscore	translation	p-value	rotation	p-value
HHS	0.013	0.9	–0.22	0.2
OHS	0.11	0.5	0.080	0.7
WOMAC	0.068	0.7	0.072	0.7
EQ-5D				
Index	–0.063	0.7	–0.21	0.3
VAS	0.062	0.7	–0.27	0.1

### Radiographic evaluations

**Figure 4.**
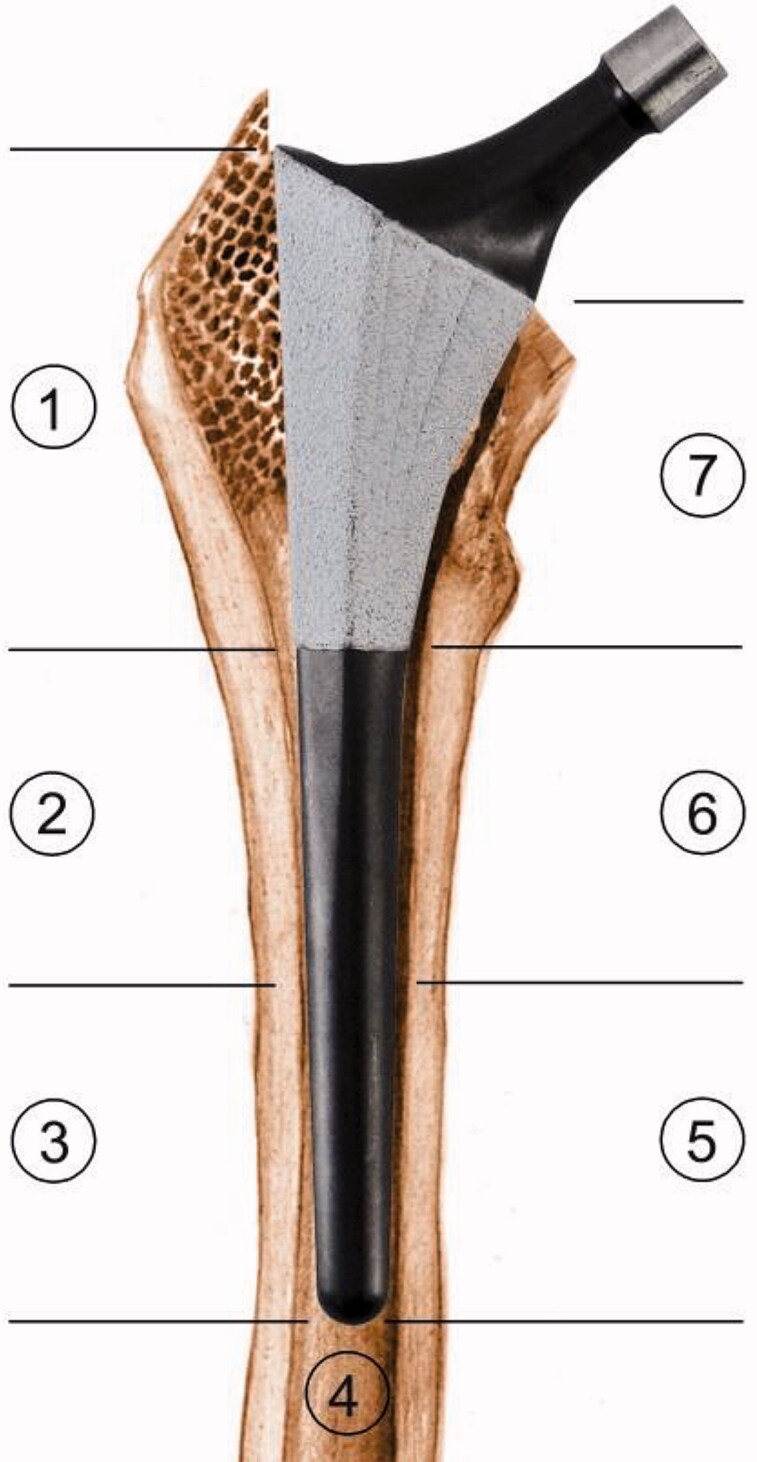
Outcomes of radiographic evaluation: number of patients per Gruen zone with cancellous hypertrophy (“spotweld formation”).

Spotweld formation was seen increasingly over time. After 2 years 35 patients showed spotweld formation in Gruen zone 2 (Figure 4, see Supplementary data). Distal reactive line formation was very rare, as it was only seen after 2 years in 3 patients in the medial and distal Gruen regions 4 till 6.

### Survival

All stems survived the complete follow-up.

### Adverse events

1 patient experienced recurrent dislocations within 4 weeks after placement of the prosthesis, for which revision of the cup was necessary. This patient was not excluded from the study, since the stem was not revised. For the final follow-up at 2 years 1 patient was excluded, because she refused to participate for personal reasons. 

## Discussion

The primary aim of this study was to evaluate migration of the Symax hip stem up to 2 years postoperatively. At 4 weeks postoperatively mean subsidence was 1.0 mm, mean retroversion was 2.4˚, and mean posterior translation was –0.4 mm. After 4 weeks the movement ceased and the prosthesis stabilized. These findings are in line with the design rationale and the coating properties of the Symax hip stem, in which early stabilization of the stem was expected to occur. Furthermore, these findings do confirm the results of the histological and histomorphometric analyses by ten Broeke et al. (2011) in which early bone ingrowth to the proximal part of the stem was shown.

Our secondary aim was to correlate Y-translation and Y-rotation at 4 weeks to clinical outcomes at 2 years. However, no clinically or statistically significant correlations were observed (all correlation coefficients were between –0.30 and 0.30). For example, the outlier patient, who showed 5.8 mm subsidence at 4 weeks, had excellent clinical outcomes for all clinical outcome parameters at 2 years. This suggests that stabilization of the stem is more important than the absolute value of migration.

Buratti et al. (2009) showed early stabilization of 85 Symax hip stems in a multicenter EBRA–FCA (Einzel-Bild-Röntgen-Analyse–femoral component analysis) study. Although subsidence increased slightly from 0.17 mm at 6 months to 0.45 mm at 2 years in their study, the threshold migration value used to define stability of a stem was 1.5 mm at 2 years. However, the accuracy of EBRA in measuring femoral stem migration is poor compared with RSA and it is less appropriate to be used as a surrogate marker for predicting long-term outcome in THA (Malak et al. 2016).

Initial migration is common for many uncemented femoral stems, and is related to initial setting of the stem in the femoral canal due to the start of postoperative weight-bearing (Campbell et al. 2011, Bøe et al. 2011, von Schewelov et al. 2012, Weber et al. 2014). However, most studies perform their first follow-up RSA analysis of uncemented hip stems only after 3 months (Bøe et al. 2011, Weber et al. 2014). In a 2-year RSA and DEXA study of the fully HA-coated Corail stem (De Puy, Warsaw, IN, USA) in patients with a femoral neck fracture, von Schewelov et al. (2012) reported that during the first 3 months the Corail stem also moved mainly distally 2.7 mm, and rotated into retroversion 3.3˚, after which the position of the prosthesis stabilized. The first RSA examination in that study was performed before weight-bearing was allowed. However, a second examination was done before the patient left hospital, which is different from our study. Campbell et al. (2011) reported the 2-year RSA results of the same Corail stem, but in patients with osteoarthritis. Initial mean subsidence in this study was 0.73 mm at 6 months, and mean retroversion was 1.8˚ at 6 months. This difference in migration for the same stem indicates that initial stability is more difficult to obtain in patients with femoral neck fractures. Bøe et al. (2011) reported in their 2-year RSA and DEXA study of the Taperloc uncemented hip stem with either the BoneMaster (BM) plasma-sprayed HA coating (both Zimmer Biomet, Warsaw, IN, USA) or the electrochemically deposited HA coating, that both stems also showed initial migration during the first 3 months and then stabilized. After 2 years mean subsidence was 0.28 mm for BM and 0.25 mm for HA, and mean retroversion was 0.46˚ for BM and 0.17˚ for HA, both of which are less compared with the results in our study. However, it is unclear whether their baseline RSA examination was performed before or after patients were allowed to weight-bear. Weber et al. (2014) showed, in their randomized controlled RSA study with 5-year follow-up, a statistically significantly larger subsidence for the collarless Furlong Active stem (JRI Orthopaedics, Sheffield, UK) (0.99 mm) compared with its precursor the collar-fitted Furlong HAC stem (0.31 mm) after 3 months. Mean retroversion was 1.2˚ for the Active stem and 0.8˚ for the HAC stem after 3 months, which is substantially less compared with the Symax stem. Their baseline RSA examination was also made before weight-bearing. No other differences in migration were seen by RSA, and after 3 months the position of both stems stabilized. These findings show that there is a substantial variability in the amount of initial stem subsidence between stem designs, as was also seen for other uncemented hip stems (Ström et al. 2007). However, as mentioned earlier, it seems that the amount of initial subsidence is less important than that the position of all these stems that stabilized after 3 months, and in our study already after 1 month. So, a minor degree of early migration within the first few months, or month, can be seen as a “settling in” period, and is not a sign of inferior osseointegration. Van der Voort et al. (2015) showed in their systematic review and meta-analysis that there was no clear pattern between early migration and late aseptic revision of uncemented femoral stems. They also suggested that the characteristic property of early stabilization of migration is probably a more suitable criterion than the absolute value of migration to identify safe uncemented hip stems. Comparison of the absolute value of migration of uncemented hip stems is not always straightforward as the postoperative reference RSA is sometimes made after the initiation of weight-bearing and sometimes before. Therefore, to create uniformity in RSA studies, we suggest performing baseline RSA examination before the start of weight-bearing, a first follow-up RSA examination after 1 month, and a third RSA examination after 3 months. The first phase (0–1 month) will show the initial stability due to the geometry of the stem. The second phase (1–3 months) will show secondary stability as a result of the osseointegrative capacity of the coating.

Excellent clinical performance of the Symax hip stem was seen, as represented in HHS, OHS, WOMAC, and EQ-5D. These findings are in line with the good clinical performance of this implant as reported in a 1-year study, in which the Symax hip stem was compared with the predominantly diaphyseal anchored Hipstar hip stem (Stryker, Duisburg, Germany) and the straight Zweymuller (SL-Plus) hip stem (Plus Orthopedics AG, Rotkreuz, Switzerland) by Bergschmidt et al. (2010). Nevertheless, they discontinued using the Symax hip stem because of subsidence of more than 10 mm in 2 patients, and 3 intraoperative periprosthetic fractures outside the study group. We did not see these complications, nor in our previous 5-year clinical and radiographic study, in which we reported excellent clinical outcomes and radiographic signs of excellent progressive proximal fixation and favorable bone remodeling (Kruijntjens et al. 2018). The cohorts of patients of both previously mentioned studies were independent. Furthermore, a Danish single-center registry study of the Symax hip stem showed a median 6.5-year survival rate of 97.5% (95% CI 96.6–98.3%), while the overall median 6.5-year survival rate for uncemented hip stems was 95% in the same Danish registry (Edwards et al. 2018). In this registry study with follow-up of up to 10 years, 29 of 1,055 hip stems were revised. No revisions were due to aseptic loosening.

A possible limitation of this study is the missing data for RSA during follow-up. The relatively high dropout rate of 10 patients out of 45 is due to the ISO criteria (ISO 16087, 2013) for stability of the markers (ME < 0.35) and for rotational reproducibility and positioning (CN < 120). Due to over-projection of markers on the medial side of the prosthesis, markers remained on the lateral side with a CN > 120. If we had used the criteria of Valstar et al. (2005), in which a CN < 150 was accepted, only 4 patients would have been excluded because of too high a condition number. Together with the patient with only 2 markers, the dropout would have been only 5 patients. However, we decided to use the stricter criteria of the ISO to decrease the error sensitivity for rotational outcomes. Patients who could not be evaluated with RSA showed excellent clinical performance on an individual basis during all follow-up evaluations.

In summary, the RSA analysis of the uncemented Symax hip stem confirms that the stem obtains stable fixation as early as 4 weeks postoperatively, after limited initial subsidence, retroversion, and slight posterior translation. There was no correlation between the amount of subsidence and retroversion at 4 weeks, and clinical outcomes after 2 years. The RSA, clinical, and radiographic evaluations all showed an excellently performing Symax hip stem after 2 years. Based on the predictive potential of the RSA technique, we anticipate excellent long-term survival of this hip stem. 

### Supplementary data

Tables 3–4 and Figure 4 are available as supplementary data in the online version of this article, http://dx.doi.org/10.1080/17453674.2019.1709956

DK: reorganized clinical database, performed calculations and statistics, wrote and revised the manuscript; LK: organised and performed RSA analysis, wrote and revised the manuscript; BK: organised and contributed to RSA analysis, revised the manuscript; LJ: performed patient inclusion and exclusion, data entry, contributed to data management, revised the manuscript; JA: contributed to study and data management, revised the manuscript; RtB: co-designed the study, included patients, operated, performed clinical and radiographic evaluations, contributed to writing, and revised the manuscript.

The authors would like to thank Jan Geurts as the second senior hip surgeon who operated on some of the patients, and to Sander van Kuijk for his help with statistical analysis.

*Acta* thanks Stergios Lazarinis and Tatu Mäkinen for help with peer review of this study.

## Supplementary Material

Supplemental Material
