# Supplementary material for: Early stabilization of the uncemented Symax hip stem in a 2-year RSA study
Source: Acta Orthop. 2020 Jan 13;91(2):159–64. doi: 10.1080/17453674.2019.1709956 (PMC7144261; doi:10.1080/17453674.2019.1709956)
Supplement: Supplemental Material [file IORT_A_1709956_SM0028.pdf]

Supplementary data

Table 3. Clinical outcomes as mean (CI) HHS, OHS, WOMAC, and EQ-5D over time

Score/subscore	Preoperatively	3 months	6 months	1 year	2 years
HHS	63 (33 to 93)	96 (82 to 110) [a]	98 (91 to 105) [a]	98 (90 to 107)	99 (92 to 105) [a]
OHS	37 (22 to 53)	23 (7 to 39) [a]	20 (6 to 34) [a]	19 (5 to 33)	18 (5 to 31) [a]
WOMAC					
Total	50 (13 to 86)	17 (–8 to 41) [a]	13 (–13 to 39) [a]	12 (–14 to 38)	10 (–14 to 34)
Pain	10 (2 to 18)	2 (–3 to 7) [a]	2 (–4 to 7)	2 (–4 to 8)	1 (–4 to 7)
Stiffness	4 (0 to 8)	2 (–1 to 5) [a]	2 (–1 to 5) [a]	1 (–1 to 4)	1 (–1 to 4)
Physical functioning	36 (10 to 62)	12 (–6 to 30) [a]	9 (–10 to 28) [a]	9 (–10 to 27)	7 (–11 to 25)
EQ-5D					
Index	69 (42 to 96)	88 (68 to 107) [a]	91 (72 to 110) [a]	91 (72 to 110)	93 (75 to 111)
VAS	67 (26 to 108)	83 (56 to 109) [a]	84 (58 to 110)	84 (55 to 113)	87 (58 to 116) [a]

[a] Indicates Wilcoxon signed-rank test p-value < 0.05 compared with the preceding clinical outcome.

Table 4. Spearman's rho correlation coefficients (CC), with p-values, for correlation between Y-translation and Y-rotation at 4 weeks and clinical outcomes (HHS, OHS, WOMAC, and EQ-5D) after 2 years

Score/subscore	CC Y translation	p-value	CC Y rotation	p-value
HHS	0.013	0.9	–0.22	0.2
OHS	0.11	0.5	0.080	0.7
WOMAC	0.068	0.7	0.072	0.7
EQ-5D				
Index	–0.063	0.7	–0.21	0.3
VAS	0.062	0.7	–0.27	0.1

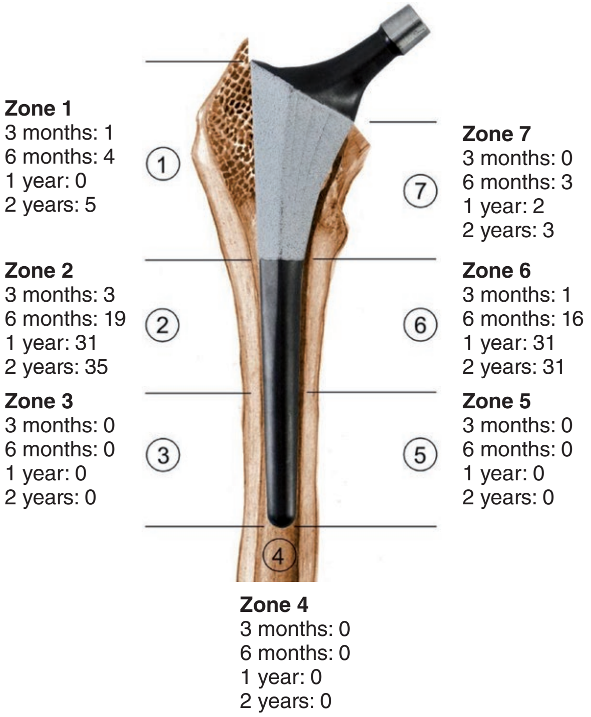

Figure 4. Outcomes of radiographic evaluation: number of patients per Gruen zone with cancellous hypertrophy ("spotweld formation").
